# Gender and Ethnicity Differences in HIV-related Stigma Experienced by People Living with HIV in Ontario, Canada

**DOI:** 10.1371/journal.pone.0048168

**Published:** 2012-12-27

**Authors:** Mona R. Loutfy, Carmen H. Logie, Yimeng Zhang, Sandra L. Blitz, Shari L. Margolese, Wangari E. Tharao, Sean B. Rourke, Sergio Rueda, Janet M. Raboud

**Affiliations:** 1 Department of Medicine, Women's College Research Institute, Women's College Hospital, Toronto, Ontario, Canada; 2 Faculty of Medicine, University of Toronto, Toronto, Ontario, Canada; 3 Department of Medicine, University Health Network, Toronto, Ontario, Canada; 4 Women's Health in Women's Hands Community Health Centre, Toronto, Ontario, Canada; 5 Department of Psychiatry, St. Michael's Hospital, Toronto, Ontario, Canada; McGill University AIDS Centre, Canada

## Abstract

This study aimed to understand gender and ethnicity differences in HIV-related stigma experienced by 1026 HIV-positive individuals living in Ontario, Canada that were enrolled in the OHTN Cohort Study. Total and subscale HIV-related stigma scores were measured using the revised HIV-related Stigma Scale. Correlates of total stigma scores were assessed in univariate and multivariate linear regression. Women had significantly higher total and subscale stigma scores than men (total, median = 56.0 vs. 48.0, p<0.0001). Among men and women, Black individuals had the highest, Aboriginal and Asian/Latin-American/Unspecified people intermediate, and White individuals the lowest total stigma scores. The gender-ethnicity interaction term was significant in multivariate analysis: Black women and Asian/Latin-American/Unspecified men reported the highest HIV-related stigma scores. Gender and ethnicity differences in HIV-related stigma were identified in our cohort. Findings suggest differing approaches may be required to address HIV-related stigma based on gender and ethnicity; and such strategies should challenge racist and sexist stereotypes.

## Introduction

HIV-related stigma remains one of the greatest barriers to the health and well being of people living with HIV (PLHIV) [1], [2]. HIV-related stigma refers to the devaluing of HIV-positive people, and may result in discrimination based on actual or perceived HIV-positive serostatus [2]. HIV-related stigma may exacerbate pre-existing social inequities based on race, class, gender, and sexual orientation [3], [4]. It is particularly important to understand the interactions of HIV-related stigma with race and gender as HIV infections are rising among women globally and there is an overrepresentation of new HIV infections among Black and Aboriginal people in Canada [5], [6].

Prior research with PLHIV has indicated that HIV-related stigma is associated with deleterious mental, psychological, and emotional health outcomes [7]–[13]. HIV-related stigma may also compromise treatment, care and support for PLHIV. Disclosure of one's HIV positive serostatus to friends, family, social support networks and health care providers has been associated with marginalization, isolation and social exclusion [12], [14], [15]. Fear of disclosure associated with purchasing and taking antiretroviral drugs may negatively impact treatment adherence [13], [16]–[18]. Thus HIV-related stigma may lead to sub-standard treatment and can also present a barrier for PLHIV accessing and retaining health care services and social supports [19]–[21]. Reducing HIV-related stigma is therefore key to promoting the health of PLHIV.

Understanding and reducing HIV-related stigma, however, is complicated by its intersection with cross-cultural differences, structural inequalities, and social processes [3], [22]–[24]. Also, there remains a gap in understanding how HIV-related stigma interacts with socio-demographic factors such as gender and ethnicity [25], [26]. In a meta-analysis of demographic correlates of HIV-related stigma among PLHIV in North America, only 2 out of 24 studies examined ethnicity and 3 examined gender [25]. This study highlighted contradictory findings regarding the associations between HIV-related stigma and race/ethnicity [25]–[29] and gender [26], [28], [30]–[34]. This area therefore warrants further exploration.

Stigma analyses are also complicated by the myriad types of stigma, including perceived, internalized, enacted, layered/compounded, and symbolic. Perceived stigma refers to awareness of negative societal attitudes, fear of discrimination and feelings of shame [7], [35]–[37]. Internalized stigma refers to an individual's acceptance of negative beliefs, views and feelings towards the stigmatized group they belong to and oneself [36], [38]–[40]. Enacted stigma encompasses overt acts of discrimination, such as violence and exclusion [35], [41]. Layered or compounded stigma refers to a person holding more than one stigmatized identity, for example a gay PLHIV who faces stigma associated with HIV as well as sexual orientation [41]–[43]. Symbolic HIV-related stigma refers to blaming and judging already stigmatized groups for causing, spreading and perpetuating the HIV epidemic [35], [44]–[46].

This study was guided by the theoretical concept of intersectionality. Intersectionality refers to the interdependent relationships between social identities (e.g. gender, race/ethnicity) and social inequities (e.g. sexism, racism) [47]–[49]. Intersectional perspectives conceptualize that the convergence of different identities (e.g. gender, race/ethnicity) produces distinct and different experiences of inequity and opportunity [47], [50]–[52]. Intersectionality has been used as a guiding theoretical framework for HIV research in the United States (U.S.) and the United Kingdom (U.K.): these studies revealed that race and gender shaped PLHIV's experiences of HIV-related stigma [53]–[55]. Qualitative research in Canada suggests that HIV prevention barriers among Black women include sexism, racism and HIV-related stigma [56]–[59], underscoring the salience of working from an intersectional approach.

The objective of this study was to understand the associations between gender, ethnicity and HIV-related stigma among PLHIV in Ontario, Canada. The secondary objective was to explore associations between HIV-related stigma and other demographic and clinical variables. A clear understanding of the associations between HIV-related stigma, gender and ethnicity can inform the development, implementation and evaluation of tailored stigma reduction interventions [41], [44], [60].

## Methods

### Ethical Considerations

Written informed consent was obtained from all participants in the OCS. This study was approved by the OHTN Governance Committee and the University of Toronto Research Ethics Board. Research methods for this study were conducted following the principles of the Helsinki Declaration.

### Study Design and Population

The study was a cross-sectional analysis using the baseline visit of the prospective observational study, the Ontario HIV Treatment Network (OHTN) Cohort Study (OCS) [61]. The OCS is a multi-site, clinical and population health prospective observational research study that recruited participants through primary and tertiary care sites. Clinical data was collected from multiple sources including electronic medical records, chart abstraction and linkages to other laboratory databases. In October 2007, the OCS introduced an annual interviewer-administered questionnaire which was either a 20-minute core questionnaire or a 90-minute extended questionnaire providing extensive socio-behavioural and demographic information [61]. For this analysis, only those completing the extended questionnaire which includes the HIV-related Stigma Scale were included. The current analysis included participants recruited from October 2007 to September 2009. Four clinic sites recruited for the OCS extended questionnaire during that time period and all were tertiary hospital clinics in Toronto. Sampling was non-random and targeted to those enrolled in a prior cohort and harder-to-reach populations including women, heterosexual men, those born in countries with a high prevalence of HIV, Aboriginal individuals, those with recent infection and injection drug users [61]. Additional detail on sampling and recruitment is available in a prior publication [61]. Refusal rates were approximately from 5% to 20% and varied by clinic [62].

Inclusion criteria for this analysis were that participants must: 1) be HIV positive based on a positive HIV antibody test or other laboratory evidence of HIV infection; 2) have provided OCS data on gender; 3) must have completed the OCS HIV-related Stigma Scale.

### Measuring HIV-related Stigma

Stigma outcomes were measured using a revised shorter version of the HIV Stigma Scale developed by Berger and colleagues [63], [64]. The 16-item stigma questionnaire has four subscales: “Personalized Stigma” (enacted stigma), “Disclosure Concerns” (enacted stigma), “Negative Self-Image” (internalized stigma), and “Concern with Public Attitudes” (perceived stigma) [63]. Each subscale contained four items on a 5-point Likert scale. The total stigma score was calculated by summing the scores for the four subscales; missing values were imputed. The total HIV Stigma score ranges from 16 to 80, with higher scores indicating a higher degree of HIV-related stigma.

### Definition of Correlates

The primary study outcomes were comparisons of severity and prevalence of HIV-related stigma by gender and ethnicity. For the comparative analyses of gender, only participants identifying as men or women were included. Due to the small number of participants (n = 9) self-identifying as transsexual, transgender or inter-sexed, these responses were excluded from the analysis.

Participants were assigned to an ethnicity category based on self-reported answer to the question “How would you describe your ethnicity?” Response options included White, Black, Latin American, South Asian, South East Asian, Arab/West Indian, Aboriginal, other, don't know, and refuse to answer. Participants were then sub-categorized into Black, White, Aboriginal, and Asian/Latin-American/Unspecified for the analyses.

Other HIV-related stigma correlates were assessed by examining associations with demographic and clinical variables including: age, HIV risk factors, sexual orientation, country of origin, immigration status, rural/urban residence, education, employment status, housing status, personal income, alcohol and drug use, duration of HIV diagnosis, CD4 count, viral load, and antiretroviral adherence.

### Statistical Analysis

Baseline demographic information, clinical characteristics, and HIV-related stigma scores were tabulated by gender (male vs. female). Categorical variables were summarized with frequencies and proportions and compared between groups using chi-square tests or Fisher's exact test. Continuous variables were summarized with medians and inter-quartile ranges (IQR) and compared between groups using Wilcoxon Rank Sum tests. Types of HIV-related stigma types were examined by tabulating the subscale scores by gender and by ethnicity within gender.

Linear regression models were used to determine the estimates of total stigma scores associated with gender and ethnicity after adjusting for other covariates. Covariates which were significant in univariate models with a significance level <0.10 or which were a priori believed to be associated with HIV-related stigma were considered as candidates for inclusion in the multivariate logistic regression model. Covariates that were considered included variables such as: age, duration of HIV infection, injection drug use (IDU), HIV risk factors, country of origin, immigration status, education, employment status, housing status, and personal income. These statistical analyses were performed using SAS Statistical Software Version 9.2 by SAS Institute Inc., Cary, NC, USA.

## Results

### Cohort Characteristics

Of the 1073 participants who completed the extended questionnaire as of November, 2009, 1035 met the inclusion criteria (38 participants were excluded as there was not enough data to calculate a total stigma score). Of these respondents, nine self-identified as transgender or inter-sexed. Seven of the nine transgender individuals were transwomen (n = 7); their median age was 46 (IQR 44–50). Cohort characteristics of the remaining 1026 participants, grouped by gender, are summarized in Table 1. There were significant differences between men and women in age, HIV risk factors, sexual orientation, ethnicity, country of origin, immigration status, years since immigration, and years since HIV diagnosis. Men were more likely to be: an older age ([median IQR] 48 years old [42]–[54] vs. 41 years old [34]–[49], p<0.0001), White (68% vs. 34%, p<0.0001), and gay/bisexual (81% vs. 5%, p<0.0001). Men also had a longer duration of HIV infection (12 years vs. 8 years, p<0.0001).

**Table 1:** Demographic and Clinical Characteristics by Gender.

Variable	Total n = 1026	Female n = 167	Male n = 859	P-value (F vs M)
Age (at time of Questionnaire)	47 (41–53)	41 (34–49)	48 (42–54)	<.0001
Risk Factor (not mutually exclusive)				
MSM	698 (68.0%)	-	698 (81.3%)	-
IDU	69 (6.7%)	10 (6.0%)	59 (6.9%)	0.68
Heterosexual contact	304 (29.6%)	161 (96.4%)	143 (16.6%)	<.0001
Blood product/Other	88 (8.6%)	21 (12.6%)	67 (7.8%)	0.04
Lesbian/Gay/Bisexual	702 (68.8%)	8 (4.8%)	694 (81.3%)	<.0001
Race/Ethnicity				
White	641 (62.5%)	57 (34.1%)	584 (68.0%)	<.0001
Black/African	170 (16.6%)	84 (50.3%)	86 (10.0%)	
Aboriginal	60 (5.8%)	8 (4.8%)	52 (6.1%)	
Asian/Latin-American/Unspecified	155 (15.1%)	18 (10.8%)	137 (15.9%)	
Country of Origin				
Canada	614 (59.9%)	58 (34.9%)	556 (64.7%)	<.0001
High HIV-prevalent country (Endemic)	195 (19.0%)	87 (52.4%)	108 (12.6%)	
Non-Endemic	216 (21.1%)	21 (12.7%)	195 (22.7%)	
Immigration Status				
Canadian-Born	614 (59.9%)	58 34.9%)	556 (64.7%)	<.0001
Canadian Citizen	305 (29.8%)	68 (41.0%)	237 (27.6%)	
Landed/Permanent Resident	72 (7.10%)	27 (16.3%)	45 (5.2%)	
Other	34 (3.3%)	13 (7.8%)	21 (2.4%)	
Years since Immigration	22 (12–32)	12 (7–22)	22 (12–37)	<.0001
Education				
Less than High School	129 (12.6%)	29 (17.4%)	100 (11.6%)	<.01
Completed High School	192 (18.7%)	35 (21.0%)	157 (18.3%)	
Some college/technical school/university	180 (17.5%)	23 (13.8%)	157 (18.3%)	
Completed college/technical school	228 (22.2%)	49 (29.3%)	179 (20.8%)	
Completed university/Post-graduate	297 (28.9%)	31 (18.6%)	266 (31.0%)	
Employment Status				
Employed FT/PT	481 (47.0%)	77 (46.1%)	404 (47.1%)	0.25
Student/Retired/Disability	454 (44.3%)	70 (41.9%)	384 (44.8%)	
Unemployed	89 (8.7%)	20 (12.0%)	69 (8.1%)	
Personal Income < $20,000/year	425 (42.2%)	81 (50.0%)	344 (40.7%)	0.03
Live in Toronto	791 (80.8%)	117 (76.0%)	674 (81.7%)	0.11
Housing				
House/Apartment/Condo	986 (96.3%)	161 (96.4%)	825 (96.3%)	0.39
Room/Housing Facility	30 (2.9%)	6 (3.6%)	24 (2.8%)	
Homeless	8 (0.8%)	0 (0.0%)	8 (1.0%)	
Alcohol/Drug Use				
Never	272 (26.5%)	66 (39.5%)	206 (24.0%)	<.0001
Monthly or less	243 (23.7%)	53 (31.7%)	190 (22.1%)	
2–4 times a month	233 (22.7%)	30 (18.0%)	203 (23.6%)	
2–3 times a week	139 (13.5%)	13 (7.8%)	126 (14.7%)	
4 or more times a week	139 (13.5%)	<6 (<3.6%)	134 (15.6%)	
Any cannabis in the last 12 months	367 (35%)	32 (19.2%)	335 (39.1%)	<.0001
Any non-medicinal drug use in the last 6 months	171 (16.7%)	13 (7.8%)	158 (18.4%)	<.01
Clinical Characteristics				
Years since HIV diagnosis	12 (6–17)	8 (5–14)	12 (6–17)	<.0001
CD4 cell count	460 (317–637)	474 (300–665)	456 (318–632)	0.76
Undetectable Viral Load	766 (74.7%)	116 (69.5%)	650 (75.7%)	0.09
Any AIDS defining condition	367 (35.8%)	64 (38.3%)	303 (35.3%)	0.45
Hepatitis B	107 (10.4%)	11 (6.6%)	96 (11.2%)	0.08
Hepatitis C	63 (6.1%)	8 (4.8%)	55 (6.4%)	3
ARV Use/Adherence				
No ARV	141 (13.8%)	37 (22.2%)	104 (12.2%)	<.001
On ARV: No missed doses	754 (73.8%)	107 (64.1%)	647 (75.8%)	
On ARV: Missed at least 1 dose in past 4 days.	126 (12.3%)	23 (13.8%)	103 (12.1%)	

*Footnote.* MSM  =  men who have sex with men, IDU  =  injection drug use, employed FT/PT  =  employed full-time/part-time, ARV  =  antiretroviral.

### Total Stigma and Subscale Scores by Gender

The total and subscale stigma scores analyzed by gender are summarized in Table 2. Women had significantly higher median total stigma scores and scores across all four subscales than men.

**Table 2:** Total Stigma Scores and Subscale Scores by Gender.

*Variables*	*Total n = 1026*	*Female n = 167*	*Male n = 859*	*P value*
Personalized Stigma (Enacted)	10 (8–14)	12 (8–16)	9 (7–14)	<.0001
Disclosure (Enacted)	15 (12–18)	16 (15–19)	15 (11–17)	<.0001
Concerns with Public Attitudes (Perceived)	13 (10–15)	15 (12–17)	12 (10–15)	<.0001
Negative Self-Image (Internalized)	15 (12–18)	16 (15–19)	15 (11–17)	<.0001
Total	49 (40–57)	56 (48–64)	48 (39–56)	<.0001

*Footnote.* Each subscale score is calculated by adding up the score from the relevant questions, with a score range of 4 – 20. The total stigma score is calculated by adding up the subscale scores, with a score range of 16 – 80. Higher scores indicate a higher degree of HIV-related stigma.

### Comparison of Stigma Scores Across Ethnicities by Gender

Among women, White women had lower median total stigma scores than Black, Aboriginal, and Asian/Latin-American/Unspecified women (Table 3). These differences were influenced by differences between ethnicities in the “Concern with Public Attitudes” subscale: Black women had significantly higher scores on this subscale than Aboriginal, Asian/Latin-American/Unspecified and White women (Table 3). There were no significant differences in the subscale scores for “Personalized Stigma”, “Disclosure Concerns”, and “Negative Self-Image” across all four ethnicities for female participants (Table 3).

**Table 3:** Total Stigma Scores and Subscale Scores by Female Gender and Ethnicity.

*Women*	*White n = 57*	*Black or African n = 84*	*Aboriginal n = 8*	*Other n = 18*	*p-value*
Personalized (Enacted) Stigma	11 (8–16)	12 (10–16)	12 (8–13.5)	10 (8–16)	0.37
Disclosure Concerns	16 (15–19)	17 (15–19)	16 (15.5–17)	16.5 (15–19)	0.79
Concern with Public Attitudes	13 (11–15)	16 (14–18)	14.8 (11.5–16)	15 (12–19)	<001
Negative Self Image	11 (9–15)	14 (10.5–16)	12 (8–17)	14 (8–15)	0.34
Total	53 (45–60)	57.5 (53–65)	55.3 (50.5–60	54.5 (47–66)	0.02

*Footnote.* Each subscale score is calculated by adding up the score from the relevant question, with a score range of 5 – 20. The total stigma score is calculated by adding up the subscale scores, with a score range of 16 – 80. Higher scores indicate a higher degree of HIV-related stigma.

Black men had significantly higher total stigma scores than Aboriginal, Asian/Latin-American/Unspecified, and White men. There were also similar significant differences across all four subscales (Table 4). Black men had the highest total stigma and the highest stigma for each subscale except Personalized Stigma, for which Aboriginal men had the highest levels.

**Table 4:** Total Stigma Scores and Subscale Scores by Male Gender and Ethnicity.

*Men*	*White n = 584*	*Black or African n = 86*	*Aboriginal n = 52*	*Other n = 137*	*p-value*
Personalized (Enacted) Stigma	9 (7–13)	11 (8–15)	12 (8–15)	10.7 (8–15)	<.0001
Disclosure Concerns	14 (11–17)	16 (12–18)	15 (11–18)	15 (12–18)	<.01
Concern with Public Attitudes	12 (10–14)	14.3 (12–16)	12.5 (10–15)	13.3 (11–16)	<.0001
Negative Self Image	10 (8–14)	11 (8–16)	10 (8.5–14.5)	11 (8–15)	<.01
Total	46 (38–54)	53.5 (42–62)	51 (40–57)	51.7 (42–60)	<.0001

*Footnote.* Each subscale score is calculated by adding up the score from the relevant question, with a score range of 5 – 20. The total stigma score is calculated by adding up the subscale scores, with a score range of 16 – 80. Higher scores indicate a higher degree of HIV-related stigma.

### Linear Regression Analysis of Total Stigma Scores

For modelling purposes, ethnicities were divided into 3 groups: White, Black and Aboriginal/Asian, Latin-American/Unspecified. Significant correlates associated with higher total stigma scores in the univariate regression analysis were female gender, non-White race, heterosexual contact or contaminated blood contact and unspecified HIV risk factor, origin from a country with high HIV prevalence, non-Canadian-born immigration status, low income, and living outside of an urban area (Toronto) (Table 5). Older age, longer duration of HIV diagnosis, lesbian gay bisexual (LGB) sexual orientation, higher education, men who have sex with men (MSM) HIV risk factor, and more frequent alcohol use, marijuana use in the last 12 months, and any non-medicinal drug use in the last 6 months were associated with lower total stigma scores in the univariate model.

**Table 5:** Univariate and Multivariable Linear Regression Models with Outcomes of Total Stigma Scores.

	*Univariate*		*Multivariable (n = 970)*	
	*Estimate*	*P value*	*Estimate*	*P value*
Gender				
Male	Reference			
Female	8.05	<.0001		
Race/Ethnicity				
White	Reference			
Black or African	8.65	<.0001		
Aboriginal/Asian, Latin-American/Unspecified	4.15	<.0001		
Gender * Race/Ethnicity				
White Male	Reference		Reference	
Black or African Male	6.19	<.0001	2.76	0.06
Aboriginal/Asian, Latin- American/Unspecified Ethnicity Male	4.19	<.0001	2.83	<.01
White Female	6.18	<.001	1.63	0.37
Black or African Female	12.27	<.0001	5.93	<.001
Aboriginal/Asian, Latin-American/Unspecified Ethnicity Female	8.40	<.001	1.83	0.45
Age (per 10 years)	−2.86	<.0001	−1.42	<.001
Risk Factor (not mutually exclusive)				
MSM	−7.97	<.0001		
IDU	0.93	0.54		
Heterosexual contact	8.30	<.0001		
Other*	4.17	<.01	3.08	0.02
Lesbian/Gay/Bisexual	−7.89	<.0001	−4.06	<.0001
Country of Origin				
Canada	Reference			
Endemic	9.09	<.0001		
Non-Endemic	3.38	<.001		
Immigrant Status				
Canadian-Born	Reference			
Canadian Citizen	5.35	<.0001		
Landed/Permanent Resident	7.81	<.0001		
Other	9.12	<.0001		
Residence				
Toronto	Reference			
Other GTA/Ontario	2.46	.01	2.57	<.01
Education				
Less than High School	Reference			
Complete HS	−2.59	0.06	−2.94	0.02
College or University	−3.76	<.01	−3.46	<.01
Employment Status				
Full/Part Time	Reference			
Student/Retired/Disability	−0.16	0.84		
Unemployed	2.81	0.05		
Room/Housing Facility/Homeless	0.08	0.97		
Personal Income < $20,000 per year	2.29	<.01		
Alcohol/Drug Use				
Alcohol Frequency				
Never or Less than Monthly	Reference			
More than Monthly	−3.06	<.0001	−1.49	0.04
Any cannabis use in last 6 months	−2.25	<.01		
Any non-medicinal drugs in last 6 months	−2.29	.02		
Clinical Characteristics				
Years since HIV diagnosis	−3.95	<.0001	−1.94	<.01
CD4 cell count (per 100 cells/mm^3^)	−0.21	0.13		
Undetectable Viral Load	−1.04	0.24		
Any AIDS defining condition	−0.21	0.79		
Hepatitis B	−0.91	0.46		
Hepatitis C	−0.83	0.60		
ARV/Adherence				
Not on ARV	Reference			
No missed doses in 4 days	−1.81	0.10		
Missed dose in last 4 days	−0.21	0.89		

*Footnote.* MSM  =  men who have sex with men, IDU  =  injection drug use, HS  =  high school, GTA  =  Greater Toronto Area, ARV  =  antiretroviral. *Other Risk Factors  =  contaminated blood contact or unspecified risk.

In the multivariate model, we examined the effect of gender and ethnicity simultaneously using an interaction term with the reference group (White male); in the final model, HIV-related scores for Aboriginal/Asian/Latin-American/Unspecified men and Black women were significantly higher than White men; and nearly significant for Black men. Residence outside of the urban area and contaminated blood contact or unspecified HIV risk factor were also associated with higher stigma scores. Correlates of lower stigma included older age, longer duration since HIV diagnosis, LGB sexual orientation, higher education level, and more frequent alcohol use (Table 5).

## Discussion

In this study female gender and non-White ethnicity were consistently associated with higher total and subscale HIV-related stigma scores. Gender and ethnicity interacted to increase the degree of stigma experienced: Black women, Aborginal/Asian/Latin-American/Unspecified men, and Black men reported the highest HIV-related stigma. Lower HIV-related stigma was associated with older age, time since diagnosis, LGB sexual orientation, higher education and alcohol frequency. Overall, findings suggest that HIV-related stigma may exacerbate certain pre-existing social inequities based on race and gender. Yet there may not be a clear-cut relationship between marginalization and HIV-related stigma: sexual minorities experienced lower HIV-related stigma than heterosexuals, and drug users reported lower rates of stigma than non-drug users.

Experiences of high levels of HIV-related stigma among women in this study, in particular Black women, suggests that sexist and racist stereotypes continue to permeate HIV discourse [65]–[69]. HIV-positive women have been positioned as “dirty, diseased and undeserving” [70] and may be blamed and shamed for HIV infection due to lasting assumptions of “deviant” sexual behaviour (e.g. sex work, promiscuity) [27], [31], [33], [60], [69], [71]. Gender norms that construct women as caregivers, mothers and nurturers can exacerbate stigma directed toward HIV-positive women who may be viewed as ill/diseased and therefore a failure in personal and social roles [32], [70], [72], [73]. The intersection of gender, race/ethnicity and class oppression are integral to understanding contexts of women's HIV risk and experiences of stigma [59], [74].

Racist stereotypes have been entrenched within constructions of HIV since the epidemic's beginning, with HIV constructed as first a “Haitian” and later an “African” disease [65], [66], [68]. HIV discourse has promoted racial stereotypes of ethnic minorities, in particular Black populations, as promiscuous, dangerous, and a threat to society [65], [68]. Higher HIV-related stigma among ethnic minorities and Aboriginal PLHIV in comparison with White PLHIV, found in this study, has also been reported in previous studies [28], [75] and reveals the embeddedness of racist stereotypes in HIV-related stigma [27]. Activism surrounding HIV and (homo)sexuality may have reduced HIV-related stigma among some LGBQ communities, yet there does not appear to be the same history of activism or success in challenging racist and sexist stereotypes ingrained in HIV discourse [65], [68]. In light of the growing HIV infection rates among Black and Aboriginal communities in Canada, in particular among Black and Aboriginal women, [5] such findings underscore the salience of challenging sexist and racist stereotypes.

Since the beginning of the epidemic, HIV and AIDS have been associated with “deviant sexuality” (e.g. homosexuality, sex work) – reinforcing the notion of the disease as punishment [65]–[68], [76]. Challenging homophobia and HIV-related stigma have been central components of lesbian, gay, bisexual, and queer (LGBQ) community mobilization and activism in North America, Western Europe and Australia since the early 1980's [68, -69]. The present study's finding that sexual minorities experience lower HIV-related stigma than heterosexuals suggests that such activism may have been successful in reducing the associations between “deviance”, homosexuality and HIV, and in creating safe places within urban LGBQ communities for PLHIV in Canada. Higher levels of HIV-related stigma among heterosexuals than sexual minorities have been reported in other studies, perhaps also due to concerns of heterosexual PLHIV being perceived as a sexual minority and/or fears of homophobia [25], [26], [45].

Drug use was associated with lower HIV-related stigma in this analyis. There are conflicting findings regarding drug use and stigma: some authors suggest drug use is a factor that exacerbates HIV-related stigma [23], [42], while others have found drug use is correlated with lower HIV-related stigma among PLHIV [32]. Drug users may have developed coping mechanisms related to drug use to decrease stigma [32], and may also perceive lower HIV-related stigma due to to greater concerns regarding drug use stigma [77].

Other factors associated with lower HIV-related stigma in the current study–urban residence, older age and higher levels of education–corroborate previous research [10], [25], [78], [79]. Factors underpinning higher stigma outside of urban areas may include a lack of visibility of HIV prevention initiatives, and thus less dialogue and education regarding HIV, reduced support services, and a lack of privacy and confidentiality [10], [78], [79]. Increased age and education may be associated with internal resources such as self-esteem, coping, life satisfaction, and emotional health, as well as higher income: these factors may mitigate experiences of HIV-related stigma [25]. Longer duration of HIV diagnosis–correlated with lower HIV-related stigma–is also associated with older age. People living with HIV may develop coping strategies and social support networks over time to help reduce stigma.

Understanding how racism and sexism may exacerbate HIV-related stigma can inform interventions to challenge stigma. Systematic reviews have underscored a paucity of evidence-based HIV-related stigma reduction interventions [23], [43], [80]. These reviews recommend multi-level interventions that use a variety of approaches. For example, individual and community interventions to reduce HIV-related stigma can incorporate: counseling and support groups for PLHIV; information and education for non-PLHIV; mass media campaigns; and skills-building for family/friends to improve care for PLHIV [23], [43], [80], [81]. Structural level interventions can include institutional protection from discrimination based on HIV serostatus, race/ethnicity, gender, and sexual orientation, as well as anti-discrimination training for health/social service providers [23], [82], [83]. HIV-related stigma reduction interventions must challenge racist and sexist stereotypes not only in HIV discourse but also in Canadian society in order to benefit women and ethnic minorities–among the most stigmatized PLHIV.

This study had several limitations. The OCS is a voluntary study involving a non-random sampling of PLHIV who are in care and there was a potential for selection bias as the participants in the OCS may differ from the general population of PLHIV in Ontario [61], [62]. It is possible that PLHIV who participate in the OCS experience lower levels of stigma and fewer concerns regarding disclosure of information. Although the sample size for the cohort was significant, the small number of female participants may have limited the determination of significant differences in stigma levels between ethnicity groups. The study was also not able to capture the experiences of transgender PLHIV due to the small number enrolled within the OCS. Small numbers of certain ethnicities such as Asian and Latin American resulted in combining these populations, precluding understanding the similarities/differences in HIV-related stigma within and between these groups. No data was available on pregnancy which may impact stigma experienced by women due to issues of disclosure or if HIV diagnosis occurred during pregnancy.

Our findings suggest the need to move beyond a layered or compounded stigma model. Layered/compounded stigma posits that more marginalized identities result in more cumulative oppression, whereas intersectionality suggests that social identities are multi-dimensional and cannot be summed up [42], [47], [48], [51], [52]. Current findings reveal that certain marginalized identitites, such as gender, may interact with HIV-related stigma to increase stigmatization while others, such as sexual orientation, may reduce experiences of HIV-related stigma. These findings build on conceptualizations of HIV-related stigma that describe racism, sexism, and pre-existing stigma towards drug users and men who have sex with men (MSM) as predisposing facilitators of HIV-related stigma [23], [43] to suggest that stigma may additionally be complicated by identity, context, history, and socio-cultural and political factors. Intersectionality therefore affords a more nuanced understanding of stigma that challenges the notion of who may be pre-disposed to stigma.

This situational nature of HIV-related stigma cannot be understated. For example, human rights violations among sexual minorities in multiple countries highlight the importance of understanding context in ascertaining the meaning and significance of sexual orientation in experiences of HIV-related stigma [84], [85]. HIV has long been constructed as a “gay disease” and an abundance of literature highlights the convergence of HIV-related stigma with sexual stigma and homophobia [32], [45], [84]–[87]. This literature serves as a reminder that the current study's findings of lower HIV-related stigma among sexual minorities may not be generalizable outstide of urban Canadian settings.

## Conclusions

In our study, we found that female gender and non-White ethnicity were associated with higher total and subscale HIV-related stigma scores. In our multivariable model, gender and ethnicity interacted to increase the degree of stigma experienced by Black women, Aborginal/Asian/Latin-American/Unspecified men, and Black men. Future research could explore why some marginalized groups may experience lower HIV-related stigma while others experience higher HIV-related stigma. Understanding contextual factors, such as culture, country, and rural/urban differences, is also central to understanding and addressing HIV-related stigma. Additional research should examine the intersection of HIV-related stigma with ethnicity, gender, and sexual orientation at multiple levels (micro, meso and macro) locally, regionally, and globally [25], [59]. There is a clear need for evidence-based interventions to challenge HIV-related stigma among diverse populations of PLHIV [43], [88], [89] at multiple levels. The complexity of HIV-related stigma necessitates engaging with all aspects of PLHIV identity to promote equity and human rights.

## References

[1] Herek GM (1999) AIDS and Stigma. Am Behav Sci 42: 1106–1116.

[2] UNAIDS (2007) AIDS epidemic update 2007. Geneva: UNAIDS. Available: http://data.unaids.org/pub/epislides/2007/2007_epiupdate_en.pdf. Accessed 2012 Jan 25.

[3] Parker R, Aggleton P (2003) HIV and AIDS-related stigma and discrimination: A conceptual framework and implications for action. Soc Sci Med 57: 13–24. 12753813 10.1016/s0277-9536(02)00304-0

[4] Sumartojo E (2000) Structural factors in HIV prevention: Concepts, examples, and implications for research. AIDS 14 Suppl: 3–10 10.1097/00002030-200006001-00002 10981469

[5] Public Health Agency of Canada (PHAC) (2009) HIV and AIDS in Canada. Surveillance Report to December 31, 2008. Ottawa: Public Health Agency of Canada. Available: http://www.phac-aspc.gc.ca/aids-sida/publication/survreport/2008/dec/pdf/survrepdec08.pdf. Accessed 25 January 2012.

[6] UNAIDS 2010. UNAIDS Report on the Global AIDS Epidemic. Geneva: UNAIDS. Available: http://www.unaids.org/globalreport/documents/20101123_GlobalReport_full_en.pdf. Accessed 2012 Jan 25.

[7] Berger BE, Ferrans CE, Lashley FR (2001) Measuring Stigma in People with HIV: Psychometric Assessment of the HIV Stigma Scale. Res Nurs Health 24: 518–529. 11746080 10.1002/nur.10011

[8] Buseh AG, Stevens PE (2007) Constrained but not determined by stigma: Resistance by African American women living with HIV. Women Health 44: 1–18. 10.1300/J013v44n03_01 17255063

[9] Courtney-Quirk C, Wolitski RJ, Parsons JT, Gomez CA (2006) Seropositive Urban Men's Study Team (2006) Is HIV/AIDS stigma dividing the gay community? Perceptions of HIV-positive men who have sex with men. AIDS Educ Prev 18: 56–67. 16539576 10.1521/aeap.2006.18.1.56

[10] Heckman TG, Anderson ES, Sikkema KJ, Kochman A, Kalichman SC (2004) Emotional distress in non-metropolitan persons living with HIV disease enrolled in a telephone-delivered, coping improvement group intervention. Health Psychol 23: 94–100. 14756608 10.1037/0278-6133.23.1.94

[11] Rueda S, Gibson K, Rourke S, Bekele T, Gardner S, et al (2011) Mastery moderates the negative effect of stigma on depressive symptoms in people living with HIV. AIDS Behav 16: 660–669. 10.1007/s10461-010-9878-6 21221755

[12] Vanable PA, Carey MP, Blair DC, Littlewood RA (2006) Impact of HIV-Related Stigma on Health Behaviors and Psychological Adjustment among HIV-Positive Men and Women. AIDS Behav 10: 473–482. 16604295 10.1007/s10461-006-9099-1 PMC2566551

[13] Ware NC, Wyatt MA, Tugenberg T (2006) Social relationships, stigma and adherence to antiretroviral therapy for HIV/AIDS. AIDS Care 18: 904–910. 17012079 10.1080/09540120500330554

[14] Abell N, Rutledge SE, Mccann TJ, Padmore J (2007) Examining HIV/AIDS provider stigma: Assessing regional concerns in the islands of the Eastern Caribbean. AIDS Care 19: 242–247. 17364405 10.1080/09540120600774297

[15] Kymla J (2005) Dynamics of hope in adults living with HIV/AIDS: A substantive theory. J Adv Nurs 52: 620–630. 16313375 10.1111/j.1365-2648.2005.03633.x

[16] Konkle-Parker DJ, Erlen JA, Dubbert PM (2008) Barriers and facilitators to medication adherence in a southern minority population with HIV disease. JANAC 19: 98–104. 18328960 10.1016/j.jana.2007.09.005 PMC2291347

[17] Rao D, Kekwaletswe TC, Hosek S, Martinez J, Rodriguez F (2007) Stigma and social barriers to medication adherence with urban youth living with HIV. AIDS Care 19: 28–33. 17129855 10.1080/09540120600652303

[18] Rintamaki LS, Davis TC, Skripkauskas S, Bennett CL, Wolf MS (2006) Social Stigma Concerns and HIV Medication Adherence. AIDS Patient Care ST 20: 359–368. 10.1089/apc.2006.20.359 16706710

[19] Bradford JB, Coleman S, Cunningham W (2007) HIV system navigation: An emerging model to improve HIV care access. AIDS Patient Care ST 21 Suppl1: 49–58. 10.1089/apc.2007.9987 17563290

[20] Naar-King S, Bradford J, Coleman S, Green-Jones M, Cabral H, et al (2007) Retention in care of persons newly diagnosed with HIV: Outcomes of the outreach initiative. AIDS Patient Care ST 21 Suppl 140–48. 10.1089/apc.2007.9988 17563289

[21] White RC, Carr R (2005) Homosexuality and HIV/AIDS stigma in Jamaica. Cult Health Sex 7: 347–359. 16864208 10.1080/13691050500100799

[22] Campbell C, Deacon H (2006) Unravelling the contexts of stigma: From internalization to resistance to change. J Community Appl Soc Psychol 16: 411–417.

[23] Mahajan AP, Sayles JN, Patel VA, Remien RH, Sawires SR, et al (2008) Stigma in the HIV/AIDS epidemic: a review of the literature and recommendations for the way forward. AIDS 22 Suppl 267–79. 18641472 10.1097/01.aids.0000327438.13291.62 PMC2835402

[24] Van Brakel WH (2006) Measuring health-related stigma: A literature review. Psychol Health Med 11: 307–334. 17130068 10.1080/13548500600595160

[25] Logie C, Gadallla TM (2009) Meta-analysis of health and demographic correlates of stigma towards people living with HIV. AIDS Care 21: 742–753. 19806490 10.1080/09540120802511877

[26] Lee RS, Kochman A, Sikkema KJ (2002) Internalized Stigma Among People Living with HIV-AIDS. AIDS Behav 6: 309–319.

[27] Lekas HM, Siegel K, Schrimshaw EW (2006) Continuities and discontinuities in the experiences of felt and enacted stigma among women with HIV/AIDS. Qual Health Res 16: 1165–1190. 17038751 10.1177/1049732306292284

[28] Emlet CA (2006) A comparison of HIV stigma and disclosure patterns between older and younger adults living with HIV/AIDS. AIDS Patient Care ST 20: 350–358. 10.1089/apc.2006.20.350 16706709

[29] Schuster MA, Collins R, Cunningham WE, Morton SC, Zierler S, et al (2005) Perceived discrimination in clinical care in a nationally representative sample of HIV-infected adults receiving health care. J Gen Intern Med 20: 807–813. 16117747 10.1111/j.1525-1497.2005.05049.x PMC1490199

[30] Steward WT, Herek M GM, Ramakrishna J, Bharat S, Chandy S (2008) HIV-related stigma: Adapting a theoretical framework for use in India. Soc Sci Med 67: 1225–1235. 18599171 10.1016/j.socscimed.2008.05.032 PMC2603621

[31] Sandelowski M, Lambe C, Barroso J (2004) Stigma in HIV-positive Women. J Nurs Scholarship 36: 122–128. 10.1111/j.1547-5069.2004.04024.x 15227758

[32] Swendeman D, Rotheram-Borus MJ, Comulada S, Weiss R, Ramos ME (2006) Predictors of HIV-related Stigma among Young People Living with HIV. Health Psychol 25: 501 – 509. 10.1037/0278-6133.25.4.501 PMC2392891 16846325

[33] Sowell RL, Lowenstein A, Moneyham L, Demi A, Mizuno Y, et al (1997) Resources, stigma, and patterns of disclosure in rural women with HIV infection. Public Health Nurs 14: 302–312. 9342922 10.1111/j.1525-1446.1997.tb00379.x

[34] Emlet CA (2005) Measuring stigma in older and younger adults with HIV/AIDS: An analysis of an HIV stigma scale and initial exploration of subscales. Res Social Work Prac 15: 291–300.

[35] Herek GM, Capitanio JP, Widaman KF (2002) HIV-related Stigma and Knowledge in the United States: Prevalence and Trends, 1991 – 1999. Am J Public Health 92: 371–377. 11867313 10.2105/ajph.92.3.371 PMC1447082

[36] Herek GM, Garnets LD (2007) Sexual orientation and mental health. Annu Rev Clin Psycho 3: 353–375. 10.1146/annurev.clinpsy.3.022806.091510 17716060

[37] Meyer IH (2003) Prejudice, social stress, and mental health in lesbian, gay, and bisexual populations: Conceptual issues and research evidence. Psychol Bull 129: 674–697. 12956539 10.1037/0033-2909.129.5.674 PMC2072932

[38] Mak WS, Poon CY, Pun LY, Cheung SF (2007) Meta-analysis of stigma and mental health. Soc Sci Med 65: 245–261. 17462800 10.1016/j.socscimed.2007.03.015

[39] Steward WT, Herek GM, Ramakrishna J, Bharat S, Chandy S, et al (2008) HIV-related stigma: Adapting a theoretical framework for use in India. Soc Sci Med 67: 1225–1235. 18599171 10.1016/j.socscimed.2008.05.032 PMC2603621

[40] Kalichman SC, Simbayi LC, Cloete A, Mthembu PP, Mkhonta RN, et al (2009) Measuring AIDS stigmas in people living with HIV/AIDS: the Internalized AIDS-related Stigma Scale. AIDS Care 21: 87–93. 19085224 10.1080/09540120802032627

[41] Nyblade LC (2006) Measuring HIV stigma: Existing knowledge and gaps. Psychology, Health & Medicine. Special Issue: Perspect Health-related Stigma 11: 335–345. 10.1080/13548500600595178 17130069

[42] Reidpath DD, Chan KY (2005) A Method for the Quantitative Analysis of the Layering of HIV-Related Stigma. AIDS Care 17: 425–432. 16036227 10.1080/09540120412331319769

[43] Sengupta S, Banks B, Jonas D, Miles MS, Smith GC (2011) HIV interventions to reduce HIV/AIDS stigma: A systematic review. AIDS Behav 15: 1075–87. 21088989 10.1007/s10461-010-9847-0 PMC3128169

[44] Deacon H (2006) Towards a sustainable theory of health-related stigma: Lessons from the HIV/AIDS literature. J Community Appl Soc Psychol 16: 418–425.

[45] Herek GM, Capitanio JP (1999) AIDS stigma and sexual prejudice. Am Behav Sci 42: 1130–1147.

[46] Herek GM, Capitanio JP, Widaman KF (2003) Stigma, social risk and health policy: Public attitudes towards HIV surveillance policies and the social construction of illness. Health Psychol 22: 533–540. 14570537 10.1037/0278-6133.22.5.533

[47] Vanable PA, Carey MP, Blair DC, Littlewood RA (2006) Impact of HIV-Related Stigma on Health Behaviors and Psychological Adjustment among HIV-Positive Men and Women. AIDS Behav 10: 473–482. 16604295 10.1007/s10461-006-9099-1 PMC2566551

[48] Collins PC (2000) Black Feminist Thought, 2nd Edition. New York: Routledge. 75p.

[49] Crenshaw K (1989) Demarginalizing the intersection of race and sex: A black feminist critique of antidiscrimination doctrine, feminist theory and antiracist politics. Univ Chicago Legal Forum 139: 139–167.

[50] Collins PH (1991) Black Feminist Thought: Knowledge, Consciousness, and the Politics of Empowerment. New York: Routledge. 109p.

[51] Shields SA (2008) Gender: An Intersectionality Perspective. Sex Roles 59: 301–311.

[52] Purdie-Vaughns V, Eibach RP (2008) Intersectional Invisibility: The Distinctive Advantages and Disadvantages of Multiple Subordinate-Group Identities. Sex Roles 59: 377–391.

[53] Collins PY, von Unger H, Armbrister A (2008) Church ladies, good girls, and locas: Stigma and the intersection of gender, ethnicity, mental illness, and sexuality in relation to HIV risk. Soc Sci Med 67: 389–397. 18423828 10.1016/j.socscimed.2008.03.013 PMC2587415

[54] Doyal L (2009) Challenges in researching life with HIV/AIDS: An intersectional analysis of black African migrants in London. Cult Health Sex 11: 173–188. 19132581 10.1080/13691050802560336

[55] Margulies P (1994) Asylum, Intersectionality, and AIDS: Women with HIV as a Persecuted Social Group. Georgetown Law J 8: 521–555. 11653125

[56] Newman PA, Williams CC, Massaquoi N, Brown M, Logie C (2008) HIV prevention for Black women: Structural barriers and opportunities. J Health Care Poor Underserved 19: 829–841. 18677073 10.1353/hpu.0.0043

[57] Williams CC, Newman PA, Sakamoto I, Massaquoi NA (2009) HIV prevention risks or Black women in Canada. Soc Sci Med 68: 12–20. 18952342 10.1016/j.socscimed.2008.09.043

[58] Tharao E, Massaquoi N, Teclom S (2006) Silent Voices of the HIV/AIDS Epidemic: African and Caribbean Women Research Study (2002–2004). Toronto: Women's Health in Women's Hands.

[59] Logie CH, James L, Tharao W, Loutfy MR (2011) HIV, gender, race, sexual orientation, and sex work: A qualitative study of intersectional stigma experienced by HIV-positive women in Ontario, Canada. PLoS Med 8: e1001124. 22131907 10.1371/journal.pmed.1001124 PMC3222645

[60] Link BG, Phelan JC (2001) Conceptualizing stigma. Annu Rev Social 27: 363–385.

[61] Rourke SB, Gardner S, Burchell AN, Raboud J, Rueda S, et al.. (2012) Cohort Profile: The Ontario HIV Treatment Network Cohort Study (OCS). Int J Epidemiol Feb 16. [Epub ahead of print]. 10.1093/ije/dyr230 22345312

[62] Raboud J, Su D, Walmsley S, Cooper C, Cohen J, et al.. (2010) Representativeness of the Ontario Cohort Study of the clinics from which they are recruiting. In Proceeding of the OHTN Research Conference, Toronto, Ontario, Canada, November 2010.

[63] Berger BE, Ferrans CE, Lashley FR (2001) Measuring stigma in people with HIV: Psychometric assessment of the HIV stigma scale. Res Nurs Health. 24(6): 518–29. 10.1002/nur.10011 11746080

[64] Gibson K, Bekele T, Rueda S (2009) Sociobehavioural Variables in the OHTN Cohort Study Extended Questionnaire: Summary of the Literature and Options for Scoring. Toronto: Ontario HIV Treatment Network.

[65] Farmer P (2001) Infections and inequalities: The modern plagues. Berkeley: University of California Press. 111p.

[66] Gilman S (1988) Disease and Representation: Images of Illness from Madness to AIDS. Ithaca, NY: Cornell University Press. 97p.

[67] Herek GEG (1988) An epidemic of stigma: Public reactions to AIDS. Am Psychol 43: 886–891. 3063145 10.1037//0003-066x.43.11.886

[68] Patton C (2002) Globalizing AIDS. Minneapolis: University of Minnesota Press. 115p.

[69] Wolffers I (2000) Biomedical and development paradigms in AIDS prevention. Bulletin of the World Health Organization 78: 267–273. 10743300 PMC2560689

[70] Lawless S, Kippax S, Crawford J (1996) Dirty, diseased and undeserving: the positioning of HIV positive women. Soc Sci Med 43: 1371–1377. 8913006 10.1016/0277-9536(96)00017-2

[71] Katz S, Nevid JS (2005) Risk factors associated with posttraumatic stress disorder symptomatology in HIV-infected women. AIDS Patient Care ST 19: 110–120. 10.1089/apc.2005.19.110 15716642

[72] Green G (1996) Stigma and social relationships of people with HIV: Does gender make a difference? In: AIDS as a gender issue: Psychosocial perspectives. Sherr L, Hankins C, Bennett L (Eds), Washington, DC: Taylor & Francis. 46–63p.

[73] Ingram D, Hutchinson SA (2000) Double binds and the reproductive and mothering experiences of HIV-positive women. Qual Health Res 10: 117 –132. 10.1177/104973200129118282 10724748

[74] Amaro H, Raj A (2000) On the margin: Power and women's HIV risk reduction strategies. Sex Roles 42: 723–749.

[75] Newman PA, Woodford MR, Logie CH (2012) HIV vaccine acceptability and culturally appropriate dissemination among sexually diverse Aboriginal peoples in Canada. Global Public Health 7: 87–100. 21390966 10.1080/17441692.2010.549139

[76] Cloete A, Simbayi LC, Kalichman SC, Strebel A, Henda N (2008) Stigma and discrimination experiences of HIV-positive men who have sex with men in Capetown, South Africa. AIDS Care 20: 1105–10. 18608067 10.1080/09540120701842720 PMC3320098

[77] Goffman E (1963) Stigma: Notes on the management of spoiled identity. Englewood Cliffs, NJ: Prentice Hall. 30p.

[78] Reif S, Golin CE, Smith SR (2005) Barriers to accessing HIV/AIDS care in North Carolina: Rural and urban differences. AIDS Care 17: 558–565. 16036242 10.1080/09540120412331319750

[79] Stephenson J (2000) Rural HIV/AIDS in the United States. JAMA 284: 167–168. 10889574

[80] Brown L, Macintyre K, Trujillo L (2003) Interventions to reduce HIV/AIDS stigma: What have we learned? AIDS Educ Prev 15: 49–69. 12627743 10.1521/aeap.15.1.49.23844

[81] UNAIDS 2005 (2005) HIV-related stigma, discrimination and human rights violations: case studies of successful programmes. Geneva: UNAIDS. Available: http://data.unaids.org/publications/irc-pub06/jc999-humrightsviol_en.pdf. Accessed 2012 Jan 25.

[82] Brondolo E, Gallo LC, Myers HF (2009) Race, racism and health: Disparities, mechanisms, and interventions. J Behav Med 32: 1–8. 19089605 10.1007/s10865-008-9190-3

[83] Schwartz J, Lindley L (2009) Impacting Sexism through Social Justice Prevention: Implications at the Person and Environmental Levels. J Pri Prev 30: 27–41. 10.1007/s10935-008-0162-8 19051035

[84] Baral S, Sifakis F, Cleghorn F, Beyrer C (2007) Elevated risk for HIV infection among men who have sex with men in low- and middle-income countries 2000–2006: A systematic review. PLoS Med 4: e339. 18052602 10.1371/journal.pmed.0040339 PMC2100144

[85] Baral S, Adams D, Lebona J, Kaibe B, Letsie P, et al (2011) A cross-sectional assessment of population demographics, HIV risks and human rights contexts among men who have sex with men in Lesotho. J Int AIDS Soc 14: 36. 21726457 10.1186/1758-2652-14-36 PMC3146892

[86] Wright K, Naar-King S, Lam P, Templin T, Frey M (2007) Stigma scale revised: Reliability and validity of a brief measure of stigma for HIV+ youth. J Adolescent Health 40: 96–98. 10.1016/j.jadohealth.2006.08.001 PMC2001277 17185215

[87] Crandall CS, Glor J, Britt TW (1997) AIDS-related stigmatization: Instrumental and symbolic attitudes. J Appl Soc Psychol 27: 95–123.

[88] Scott-Sheldon LA, Kalichman SC, Carey MP, Fielder RL (2008) Stress management interventions for HIV+ adults: a Meta-analysis of randomized controlled trials, 1989 to 2006. Health Psychol 27: 129–39. 18377131 10.1037/0278-6133.27.2.129 PMC2409585

[89] Simbayi LC, Kalichman S, Strebel A, Cloete A, Henda N, et al (2007) Internalized stigma, discrimination, and depression among men and women living with HIV/AIDS in Capetown, South Africa. Soc Sci Med 64: 1823–31. 17337318 10.1016/j.socscimed.2007.01.006 PMC4271649

